# Comparison of Plasma Deposition of Carbon Nanomaterials Using Various Polymer Materials as a Carbon Atom Source

**DOI:** 10.3390/nano12020246

**Published:** 2022-01-13

**Authors:** Alenka Vesel, Rok Zaplotnik, Gregor Primc, Domen Paul, Miran Mozetič

**Affiliations:** 1 Department of Surface Engineering, Jozef Stefan Institute, Jamova cesta 39, 1000 Ljubljana, Slovenia; rok.zaplotnik@ijs.si (R.Z.); gregor.primc@ijs.si (G.P.); domen.paul@ijs.si (D.P.); miran.mozetic@ijs.si (M.M.); 2 Jozef Stefan International Postgraduate School, Jamova cesta 39, 1000 Ljubljana, Slovenia

**Keywords:** carbon nanowalls, plasma deposition, fast synthesis, one-step procedure, polymer source

## Abstract

Carbon nanowalls are promising materials for various electrochemical devices due to their chemical inertness, desirable electrical conductivity, and excellent surface-to-mass ratio. Standard techniques, often based on plasma-assisted deposition using gaseous precursors, enable the synthesis of top-quality carbon nanowalls, but require long deposition times which represents a serious obstacle for mass applications. Here, an alternative deposition technique is presented. The carbon nanowalls were synthesized on titanium substrates using various polymers as solid precursors. A solid precursor and the substrate were mounted into a low-pressure plasma reactor. Plasma was sustained by an inductively coupled radiofrequency discharge in the H-mode at the power of 500 W. Spontaneous growth of carbon nanomaterials was observed for a variety of polymer precursors. The best quality of carbon nanowalls was obtained using aliphatic polyolefins. The highest growth rate of a thin film of carbon nanowalls of about 200 nm/s was observed. The results were explained by different degradation mechanisms of polymers upon plasma treatment and the surface kinetics.

## 1. Introduction

For decades, the synthesis of carbon nanomaterials has been a hot topic of interdisciplinary research because of the numerous possible applications. The materials exhibit good chemical inertness, reasonable electrical conductivity, and a large surface-to-mass ratio. A variety of carbon nanomaterials have been invented, including fullerenes, nanotubes, nanowires, graphene and its derivatives, and less-oriented materials such as soot. Of particular importance are carbon nanomaterials in the form of evenly distributed flakes stretching from a substrate surface. Such materials are promising for application in electrochemical and photoelectrical devices such as supercapacitors, super-batteries, fuel cells, and photo-catalyzers [1,2,3,4,5,6]. A sophisticated version of such materials is carbon nanowalls (CNWs). Carbon nanowalls are flakes of multilayer graphene sheets vertically oriented on a suitable substrate. The typical thickness of the CNWs is several nm, and the area of a flake is of the order of µm^2^. The distance between neighboring flakes in carbon nanowalls is several 10 µm, thus making this material superior in terms of the surface-to-mass ratio.

Several methods were proposed for the synthesis of carbon nanowalls. They are usually based on classical chemical vapor deposition (CVD) or plasma-enhanced chemical vapor deposition (PECVD). These techniques usually require a long deposition time of minutes or even hours. The current progress in the synthesis of carbon nanowalls has been reviewed in the papers [7,8]. Recently, we have shown that nitrogen-containing carbon nanomesh can be deposited in nitrogen plasma using polymer polyethylene terephthalate as the source of a carbon precursor [9]. The aim of this paper was to investigate the influence of the type of polymer material used as a carbon precursor on the growth of carbon nanostructures. Therefore, several different polymers were used in the study presented in this paper. Additionally, besides the effect of the source of the carbon precursor on the CNWs’ growth, the effect of the type of processing plasma gas was also investigated. 

## 2. Materials and Methods

### 2.1. Plasma Synthesis of Carbon Nanomaterials

**Figure 1:**
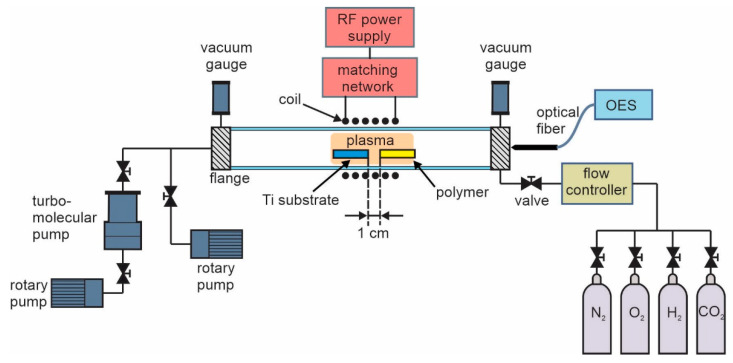
Experimental set-up.

The deposition of carbon nanomaterials was performed in an inductively coupled radiofrequency plasma (ICP-RF) system shown in Figure 1. The system consisted of the discharge tube with a coil of six turns that was positioned in the middle of the tube. The tube was made of borosilicate glass with dimensions 80 cm in length and 4 cm in diameter. The system was first pumped with a Hi-Cube 300 Classic pumping station (Pfeiffer Vacuum, Asslar, Germany), consisting of a turbomolecular pump of a nominal pumping speed of 260 L/s backed with a rotary pump with a nominal pumping speed of 5 m^3^/h. This pumping station was used to get the base pressure of 10^−2^ Pa and to achieve low levels of the residual atmosphere. After achieving the base pressure, we continued pumping only with a rotary pump with a nominal pumping speed of 80 m^3^/h. A gas of commercial purity was released into the discharge system using a mass flow controller Aera FC7700 (Advanced Energy, Denver, CO, USA). Various gasses were used to manipulate the morphology and chemical composition of deposited carbon nanostructures. The deposition was thus performed using N_2_ (43 sccm), O_2_ (35 sccm), H_2_ (82 sccm), and CO_2_ (58 sccm), keeping the gas pressure constant at 16 Pa. This was the pressure obtained using the smallest flow rate that was possible to set on our flow controller. If the flow rate was higher, it was not good for the synthesis because the polymer degradation products were too quickly pumped away. Here we should mention that CNWs could also be synthesized in a closed system without pumping, but this was not practical because it was more difficult to control the constant experimental conditions and the repeatability.

Deposition of carbon nanomaterials was performed on the substrates made from titanium foil which were placed in the middle of the coil (i.e., in the glowing discharge). Ti substrates were in the form of a square with a size of 8 × 8 mm^2^. At the same time, a piece of a polymer material was also placed into the discharge tube, as shown in Figure 1. 

The polymer was placed 1 cm before the Ti substrate. The polymer material served as a source of carbon precursors. Different polymer materials (from Goodfellow Ltd., Huntingdon, UK) were used to study the influence of the source material on the properties of the nanocarbon deposit. The following polymer materials were selected: low-density polyethylene (LDPE), high-density polyethylene (HDPE), polypropylene (PP), polyamide (PA6), polyethylene terephthalate (PET), polystyrene (PS), and acrylonitrile butadiene styrene (ABS). For all polymers, equal masses (200 mg) were used. The amount of a polymer does not affect the synthesis of CNWs as long as it is large enough to provide a sufficient flux of carbon precursors to the polymer surface. We found that 200 mg of a polymer is more than enough, so we always chose this mass of a polymer. If the amount of a polymer was too small compared to the size of the substrate surface, the surface would be poorly covered with CNWs because of the insufficient flux of carbon precursors.

The deposition time was varied using PP polymer to find the most optimal time for the deposition of CNWs—which was 60 s. Then, the deposits were formed using all the above-mentioned polymers at a constant treatment time of 60 s. 

The plasma was ignited and sustained at the forward power of the RF generator of 500 W (Advanced Energy, Denver, CO, USA). The RF generator operated at the standard industrial frequency of 13.56 MHz. Inductively coupled plasma was sustained in the H-mode, where the absorbed power was high (reflected power only 20 W). At these conditions, a Ti substrate was heated in the plasma because of exothermic heterogeneous surface reactions and reached a temperature of approximately 800 °C after several seconds. At the same time, the polymer material was also heated and melted. Degradation products of polymer evaporated from the solid precursors and deposited on the Ti substrate forming a layer of nanowalls. 

To understand deposition mechanisms occurring in plasma when using various gases and polymer materials, we characterized the plasma by optical emission spectroscopy (OES) using AvaSpec-3648 Fiber Optic Spectrometer (Avantes, Apeldoorn, The Netherlands). The spectrometer resolution was 0.5 nm in the range of wavelengths between 200 to 1100 nm. The integration time was 1 ms. The spectrometer was placed at the end of the tube on its axis because the sidewalls of the tube quickly became opaque due to the formation of a deposit.

### 2.2. Characterization of the Samples

The surface morphology of the deposits was analyzed by secondary electron microscopy (SEM). Microscopic images were acquired in immersion mode using Schottky field emission scanning electron microscope with a monochromator (Thermo Fisher Verios 4G HP, Waltham, MA, USA).

The chemical composition of the samples was analyzed by X-ray photoelectron spectroscopy (XPS). The characterization was performed by using an XPS (TFA XPS Physical Electronics, Münich, Germany). The samples were excited with monochromatic Al Kα_1,2_ radiation at 1486.6 eV over an area with a diameter of 400 µm. Photoelectrons were detected with a hemispherical analyzer positioned at an angle of 45° with respect to the normal of the sample surface. Survey spectra were measured to determine the surface composition—i.e., the presence of any other elements except carbon. The survey spectra were measured at a pass energy of 187 eV with an energy step of 0.4 eV. The measured spectra were analyzed using MultiPak v8.1c software (Ulvac-Phi Inc., Kanagawa, Japan, 2006) from Physical Electronics, which was supplied with the spectrometer. Standard sensitivity factors were used for the calculation of the surface composition.

## 3. Results and Discussion

### 3.1. Influence of the Deposition Time

**Figure 2:**
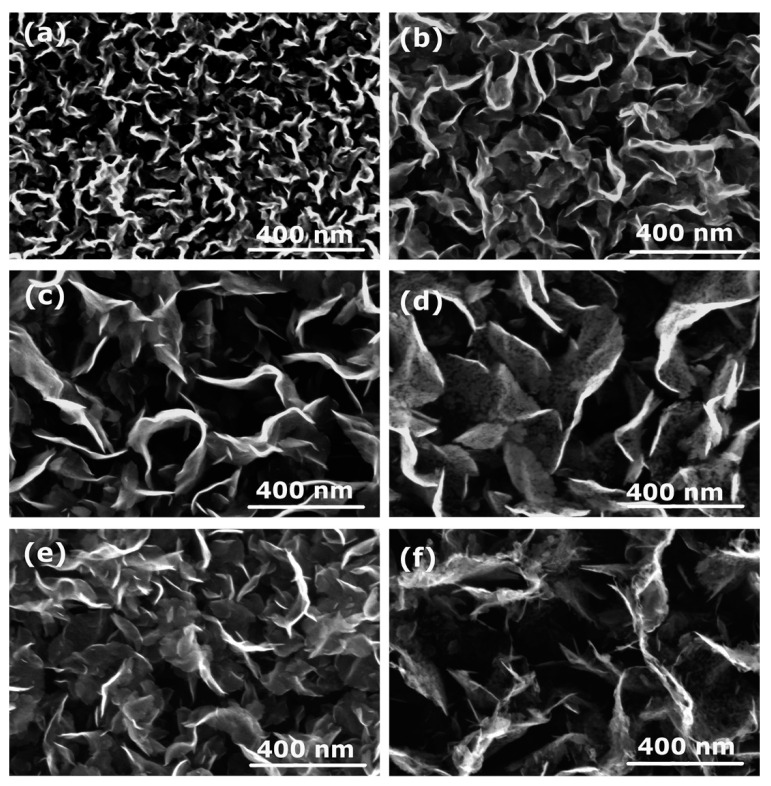
SEM images of CNWs for various deposition times in N_2_ plasma when using PP polymer: (**a**) 10 s, (**b**) 20 s, (**c**) 30 s, (**d**) 60 s, (**e**) 90 s, and (**f**) 120 s.

Various deposition times were used to find the most optimal conditions for the synthesis of a thin film of carbon nanowalls. In Figure 2, examples of SEM images of CNW deposits when using the PP polymer as a source of carbon atoms are shown. One can observe that at the shortest deposition time of 10 s, CNWs are small and dense on the substrate surface (Figure 2a). When the deposition time is increased, the size of the vertical flakes increases, as well as the distance between them (Figure 2a–c), reaching the maximum size of the nanowalls and a maximum distance between them at 60 s of treatment (Figure 2d). At longer deposition times (Figure 2e,f), CNWs become slightly smaller and denser again. Furthermore, the edges of CNWs are less sharp, indicating that etching and their destruction had already occurred. 

**Figure 3:**
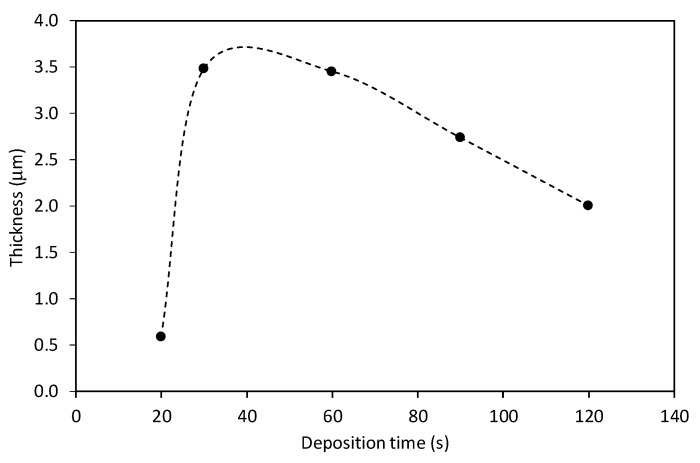
The thickness of CNWs, shown in Figure 2, versus deposition time.

The results presented in Figure 2 are further evidenced in Figure 3, which represents the thickness of the CNW film versus the deposition time. The deposition time does not affect just the morphology of CNWs but also the thickness of the deposited layer. One can observe a maximum thickness at the treatment times between 30 and 60 s. A decrease in the film thickness at a longer treatment time is a consequence of plasma etching and simultaneous removal of the deposit during its growth. 

The results presented in Figure 2 and Figure 3 are in agreement with our previous investigation, where we deposited CNWs in nitrogen plasma with the same experimental conditions but using PET polymer as the solid precursor and found the most optimal deposition time was 60 s [9]—i.e., the same as in this study. Therefore, in all further experiments, the deposition time was fixed to 60 s.

### 3.2. Influence of the Polymer Material as a Carbon Atom Source

In the next set of experiments, different polymer materials were used to study the influence of the carbon source material on the characteristics of the deposits. Therefore, examples of aromatic (PET, PS, ABS) and aliphatic (LDPE, HDPE, PP, PA6) polymers were used to produce different plasma radicals acting as building blocks for the growth of CNWs. Some of these polymers were olefins (PS, LDPE, HDPE, PP), whereas the others contained heteroatoms O and/or N (PET, ABS, PA6). The structure of these polymers is shown in Table 1.

**Figure 4:**
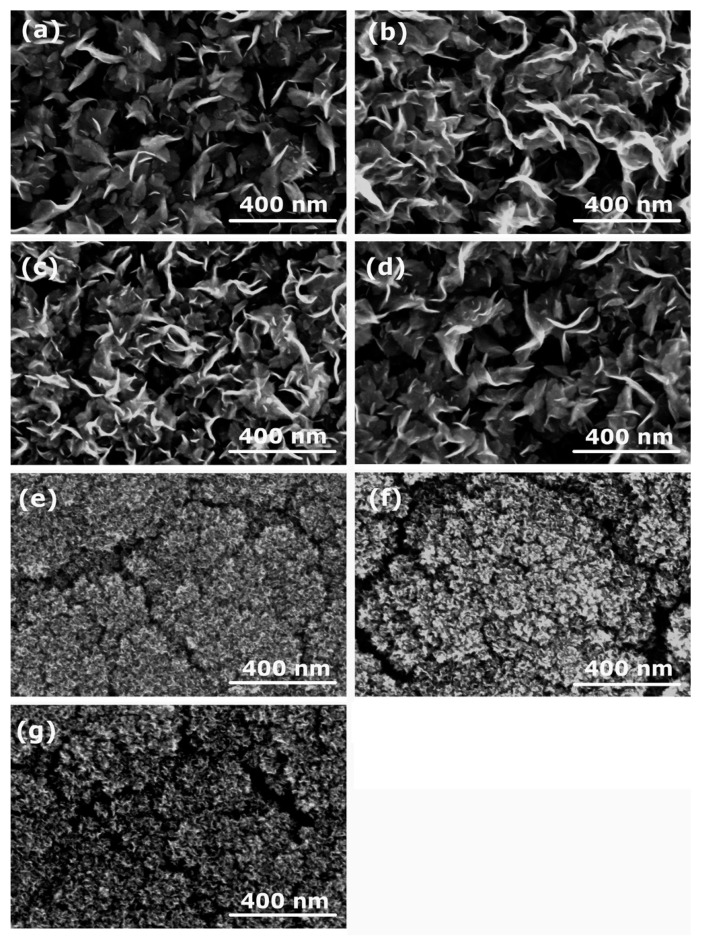
SEM images of carbon nanostructures synthesized from various polymers: (**a**) PP, (**b**) PA6, (**c**) LDPE, (**d**) HDPE, (**e**) PET, (**f**) PS, and (**g**) ABS. Deposition time in nitrogen plasma was 60 s.

SEM images of the deposits synthesized from the above-mentioned polymers are shown in Figure 4. Figure 4a–d show the deposits formed on the titanium substrate when using polymers PP, PA6, LDPE, and HDPE. In all four cases of polymers, we can clearly observe the formation of CNWs. However, the next three images shown in Figure 4e–g significantly differ from Figure 4a–d. Instead of CNWs, a dense mesh of small nanocarbon was formed, resembling cauliflower-like structures. These samples with the cauliflower-like structures were synthesized from polymers PS, PET, and ABS, all examples of aromatic polymers. In contrast, images with CNWs (Figure 4a–d) were all obtained when using aliphatic polymers as a source of the carbon precursor. The results shown in Figure 4 indicate that there must be a significant difference in the thermal degradation of aromatic and aliphatic polymers upon plasma conditions leading to different carbon radicals acting as building blocks for CNWs. Aromatic polymers are in general more thermally stable than aliphatic, and it seems that aromatic rings, which are in the first approximation similar to a graphite structure (except that the rings are terminated with hydrogen), are not key building blocks for the growth of CNWs, or they have a different influence on the formation of the nucleation sites and growth process [10]. In addition, the dynamics of the migration and reorientation of these species as well as the time scale of their growth may be different, thus affecting the growth of the deposit.

To get a further insight into the degradation mechanisms of various polymers and the consequent formation of their degradation products, we studied the literature. The explanation is far from being simple because of the presence of various factors in plasma (i.e., heat load, irradiation, reactive (oxidizing) plasma species) that may all influence the degradation kinetics of polymers in plasma. Depending on the environmental conditions, polymers can undergo different degradation mechanisms [11]: (i) thermal degradation, (ii) photochemical degradation, and (iii) oxidative degradation. Thermal degradation is caused by a temperature increase leading to conformational changes, bond dissociation, and radical formation. Photochemical degradation is triggered by photon irradiation (visible, UV, VUV), causing bond dissociation and cross-linking. If the treatment is performed in an oxidizing atmosphere such as air, the bond-scission is followed by oxidation (photooxidative degradation). Oxidative degradation is caused by the presence of oxygen. This process is particularly effective in gaseous discharges (i.e., oxygen plasmas) that are a rich source of various reactive oxygen species—the most important are neutral oxygen atoms. Even when using discharges sustained in other gasses, some oxygen is present in vacuum systems as water vapor. In the presence of reactive oxygen species, the process of polymer degradation is often initiated by hydrogen subtraction from polymer, followed by various reactions [12]. However, plasmas are not only a rich source of oxygen atoms but also a source of radiation by photons (UV/VUV). Furthermore, because of exothermic heterogeneous surface recombination and chemical reactions of oxygen radicals on the polymer surface, the polymer can be heated well above the melting temperature. All the above-mentioned processes occur on the polymer surface upon plasma conditions simultaneously, making the plasma–polymer interaction very complex. In extreme cases of polymer oxidation, polymer burning occurs [11]. The interior of the polymer is subjected to thermal degradation, causing the formation of molecular fragments, which migrate towards the polymer surface, where they mix with oxygen and burn [11]. When using plasma afterglows for surface functionalization of polymers, the polymer is kept at room temperature. Therefore, only prolonged treatment with oxygen radicals can cause etching and degradation. In our case, the polymer was placed in the intense glowing region; therefore, it was heated above its melting temperature in a short time. Furthermore, experiments were performed in nitrogen plasma with a low base pressure; therefore, the contribution of oxidizing species to polymer degradation is negligible in our case. For this reason, we can assume that thermal degradation is the most important mechanism in our conditions. For all polymers probed in this study, the final degradation product after prolonged plasma treatment was black carbon residues, although some visual differences were observed during the plasma treatment. ABS polymer immediately carbonized. PS and PET polymers shrank into a ball, started boiling, and finally carbonized. LDPE and HDPE twisted, intensely boiled, and then carbonized. PA6 was also intensively boiling, but it neither shrank nor twisted. For PP polymer, no special visible changes were observed in terms of twisting, shrinkage, or bubble formation, and it looked as if only evaporation was occurring on the surface before the carbonization. From these observations, it seems that intensive boiling or evaporation is the most important process for providing appropriate volatile low-mass species, which served as building blocks of nanocarbon on the titanium substrate. 

To further check whether just high temperature causing thermal degradation is sufficient for CNW synthesis or whether plasma is needed at all, we performed an additional experiment in the same vacuum system without igniting the plasma. An additional heater was used to heat the polymer and the Ti substrate; however, CNWs did not form. In yet another modification of this experiment, the Ti substrate was placed in the glowing plasma, whereas the polymer was placed far away from plasma and heated by an additional heater. In these conditions, CNWs were formed on the Ti-substrate, thus proving that plasma has an important role in the growth of CNWs on the substrate or in the modification of polymer-degradation products reaching the plasma. However, for polymer degradation itself, the plasma is not essential for the deposition of nanocarbon on the titanium surface; therefore, a simple thermal degradation can also be applied.

Polymers may have different weight-loss rates during their thermal degradation [13]. Furthermore, different degradation products may also be formed. As found in the literature review, the most important thermal and photodegradation products of the polymers used in our study are summarized in Table 2 [14,15,16,17,18,19]. We can notice that in the case of aromatic polymers, degradation products contain aromatic rings (e.g., styrene monomer), whereas in the case of aliphatic polymers, various low-molecular unsaturated and saturated aliphatic compounds are formed. As already mentioned before, despite some similarities of aromatic rings to graphite structure, it is apparent that aromatic rings are not important building blocks in the formation of CNWs. It is much more likely that low-molecular aliphatic compounds that can be further dissociated in plasma are the most important building blocks. This is also in agreement with the classical PECVD techniques that apply CH_4_, C_2_H_2,_ or even ethanol and hexane vapor or fluorinated compounds CF_4_, CHF_3_, and C_2_F_6_ for the deposition of CNWs [20,21,22]. Currently, it is supposed that C_2_ dimers are the most important building units for CNWs growth [23]. From C_2_ dimers, higher mass-carbon clusters C_n_H_x_^+^ may be formed, initiating the growth process [23,24]. 

In fact, we have found only one publication, published by Lehman et al. [24], where authors used aromatic precursor p-xylene for the synthesis of CNWs. They also used ICP-RF plasma but with a very low discharge power of just 150 W. Very low power was responsible for breaking the C–H bond in the methyl group attached to the aromatic ring, leaving the aromatic ring (which exhibits higher stability) rather intact. The growth of CNWs was explained by the condensation of p-xylyl radicals and the formation of polycyclic aromatic 2,6-dimethylanthracene. In another publication by Hsu et al. [25], the authors also reported the synthesis of CNWs using a low-power plasma (60 W) and 1,2-dichlorobenzene as the precursor; however, methane was also introduced into the plasma to enhance the growth process. Since methane is a commonly used precursor, it is questionable if 1,2-dichlorobenzene was needed for the successful deposition of CNWs at all. In RGA spectra, the authors found substantial amounts of C_2_ together with smaller amounts of C_6_ and C_6_H_6_. 

**Figure 5:**
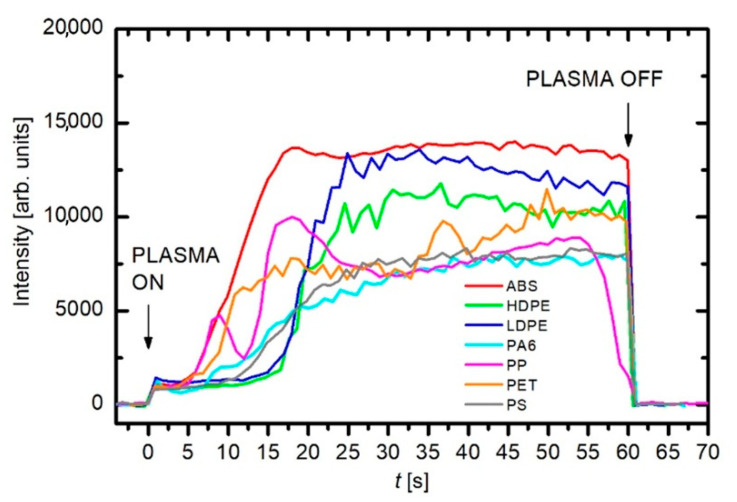
Variation of the OES intensity of C_2_ species formed during burning of different polymer materials in nitrogen plasma.

The formation of C_2_ was also monitored in our experiment using OES. The evolution of C_2_ species during the plasma treatment of various polymers is shown in Figure 5. No correlation was found between the intensity of C_2_ species and the morphology of the deposits. Nevertheless, the final growth of nanostructures obviously depends on many factors: supply of appropriate building units, their consumption by the growth process, the time scale needed for transport and reorganization, etc. These processes do not depend just on the type of precursor used but also on the surface temperature, which in turn depends on the discharge power [26]. Building units are not produced only as a consequence of the thermal decomposition of polymers, but they can also be produced in plasma as well as within the sheath, where fluxes of ions may be important, especially at the initial stages of the formation of nucleation sites [26]. 

**Table 2:** Thermal and photooxidative degradation products. Information for melting and deposition temperature was obtained from [16].

Polymer	Melting T	Decomposition T	Thermal Degradation Products	Refs.
PS	503 K	549 K	monomer styrene (40%), dimer, trimer, tetramer, pentamer, benzene, ethylbenzene, α-methylstyrene; in the presence of oxygen: phenol, ketones, benzoic acid, benzyl alcohol, benzaldehyde	[14,15,16]
PE	378–408 K	650 K	propene (up to 25%), propane, ethene, ethane, butene, hexene (formation of the transition state six-membered ring)	[14,15,16]
PP	443 K	624 K	pentane (24%), 2-methyl-1-pentene, 2,4-dimethyl-1-heptene, propane	[14,15,16]
PET	523–533 K	698 K	cleavage of ester groups and formation of carboxylic acids and vinyl esters (benzoic acid (43%), acetaldehyde (16%), CO_2_, vinyl esters of benzoic acid), anhydride containing oligomers, cyclic oligomers; also scission through a six-membered ring transition state; formation of non-volative residues of interconnected aromatic rings was also reported.	[15,16,17,18]
PA6	498–508 K	708 K	cyclic oligomers, caprolactam (73%), CO_2_	[19]
ABS	383–398 K	693 K	degradation to its constituents, depending on temperature: butadiene, sytrene, ethylbenzene, N-containing products	[27,28]

**Figure 6:**
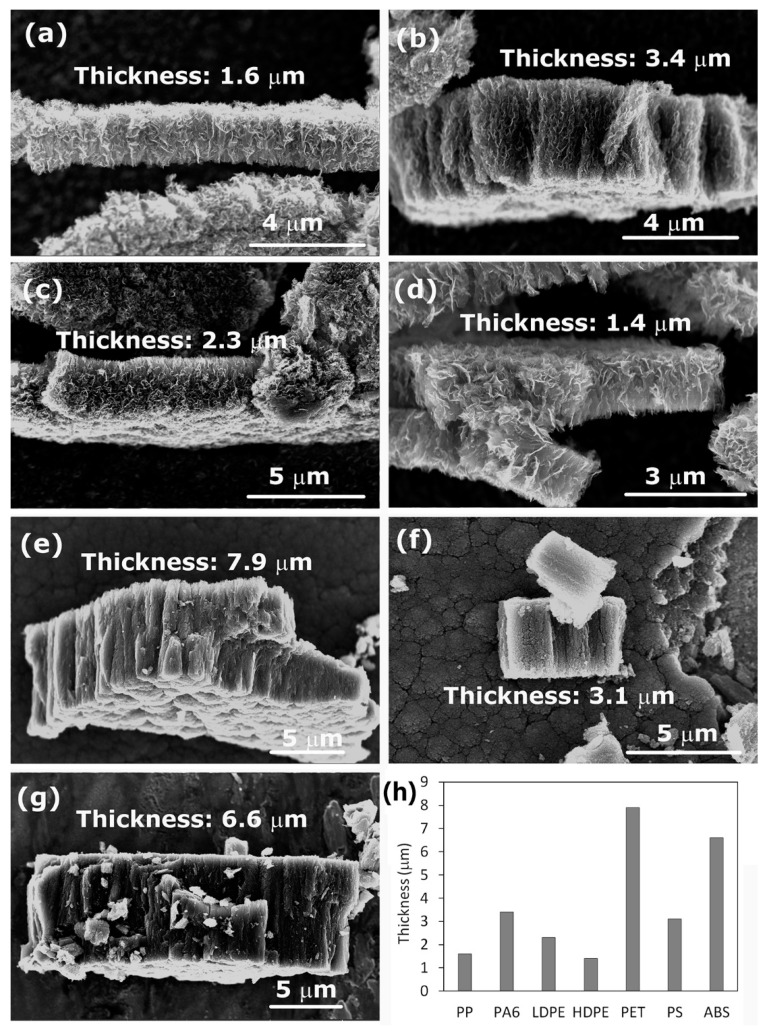
Cross-section of the deposited carbon nanostructures: (**a**) PP, (**b**) PA6, (**c**) LDPE, (**d**) HDPE, (**e**) PET, (**f**) PS, (**g**) ABS, and (**h**) a thickness of the deposited layer. Deposition time was 60 s.

Here we should also note that the different morphology of the deposited carbon nanostructures when using aromatic or aliphatic polymer precursors is not the only outcome of this study. We have also noticed significant variations in the thickness of the deposited layers. In Figure 6a–g cross-sections of the deposits and their thicknesses are shown. In general, we can observe that, when using aromatic polymers (Figure 6e–g), the layers are thicker than for aliphatic polymers (Figure 6a,c,d). It was reported that etching and removal of amorphous carbon from the deposit was an important step in the formation of CNWs [29]. In addition, hydrogen was found to play a significant role in the growth of CNWs [30,31,32]. A generally accepted mechanism of CNW growth includes: (i) adsorption of CH_x_ radicals and formation of an amorphous layer, (ii) formation of defects acting as nucleation sites, and (iii) migration and nucleation of carbon species leading to the growth of graphene sheets [29,33]. Therefore, etching is essential for helping the formation of appropriate nucleation sites and removal of small randomly oriented structures, including amorphous fractions, thus enabling the growth of relatively large vertical graphene sheets. Moreover, etching also prevents the formation of additional graphene layers by the removal of weakly bonded carbon atoms, and it enhances the migration of carbon precursors [33]. All these facts can explain the formation of thicker layers when using aromatic polymers. It seems that there was no simultaneous etching of amorphous parts during the growth to allow the migration, nucleation, and formation of highly ordered structures. Furthermore, simple low-weight polymer degradation products in the case of aliphatic compounds probably have a higher ability for migration and appropriate orientation during CNW growth than larger aromatic fractions. 

### 3.3. Influence of the Processing Gas

As mentioned in the previous subsection, the presence of oxidative species and radiation may influence the thermal degradation mechanisms of polymers. However, it was also reported that the addition of hydrogen, argon, and even oxygen in classical PECVD techniques using a CH_4_ precursor could greatly improve the quality of CNWs [7]. The quality of carbon nanowalls is often attributed to the quantity of inadequately bonded carbon. The best quality is attributed to graphene sheets free from the amorphous hydrogenated carbon phase. The amorphous phase will deposit, especially at elevated pressures, but will be effectively removed by weak bombardment with positive ions and chemical interaction with reactive neutral species such as H and/or O atoms. Some authors also performed the synthesis of CNWs in nitrogen or ammonia plasma to allow for the doping of CNWs with nitrogen atoms and thus affecting their electronic properties [8]. This was also one of the reasons for choosing polymers such as PA6 and ABS in this investigation, because they contain nitrogen. We wanted to check if this can help obtain CNWs with more N-doped atoms. Table 3 shows the surface composition of the deposits as revealed from XPS survey spectra acquired on samples whose SEM images are shown in Figure 4. We have to stress again that the deposits were formed using nitrogen plasma. The measured concentration of nitrogen for all polymers is rather small. The highest amounts were found when using PS, PET, and PA6 with no significant difference between them. For ABS polymer, the nitrogen content was at the detection limit of XPS. Therefore, using N-containing polymer as a carbon as well as a nitrogen precursor does not allow a higher content of nitrogen to be obtained within the surface film as probed by XPS. 

**Figure 7:**
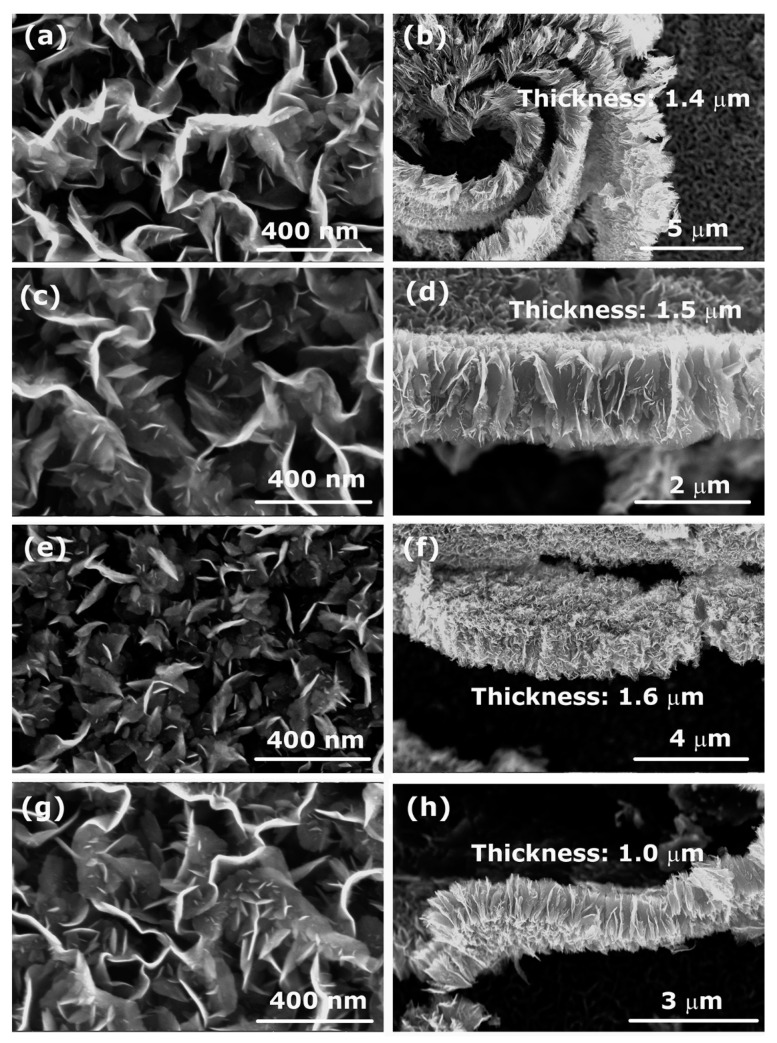
SEM images of CNWs (left) and their cross-section (right) synthesized from the aliphatic PP polymer in plasma created in: (**a**,**b**) O_2_, (**c**,**d**) H_2_, (**e**,**f**) N_2_, and (**g**,**h**) CO_2_ gas. Deposition time was 60 s.

The influence of the processing gas on the growth and morphology of CNWs was studied using different gases instead of nitrogen to reveal the role of the presence of oxygen, hydrogen, or other species in the plasma. CNWs were synthesized from PP and PS polymers using plasmas created in H_2_, O_2_, N_2_, and CO_2_ gases. Figure 7a–h shows SEM images of the deposits (left column) and their cross-sections (right column) for the case of aliphatic PP polymer, whereas Table 4 shows the surface compositions as deduced from XPS survey spectra. An important conclusion from Figure 7 is that no matter what gas we use, the CNWs are always synthesized on the surface when using this aliphatic polymer as the solid precursor. This is another indication that, in our case, oxidative degradation of a polymer is not the most important mechanism that leads to the growth of CNWs, at least for this type of polymer. The CNWs only differ in the size of the graphene flakes. Furthermore, their chemical composition (Table 4) is similar—according to experimental error, the oxygen concentration is practically the same, regardless of the type of processing gas used.

**Figure 8:**
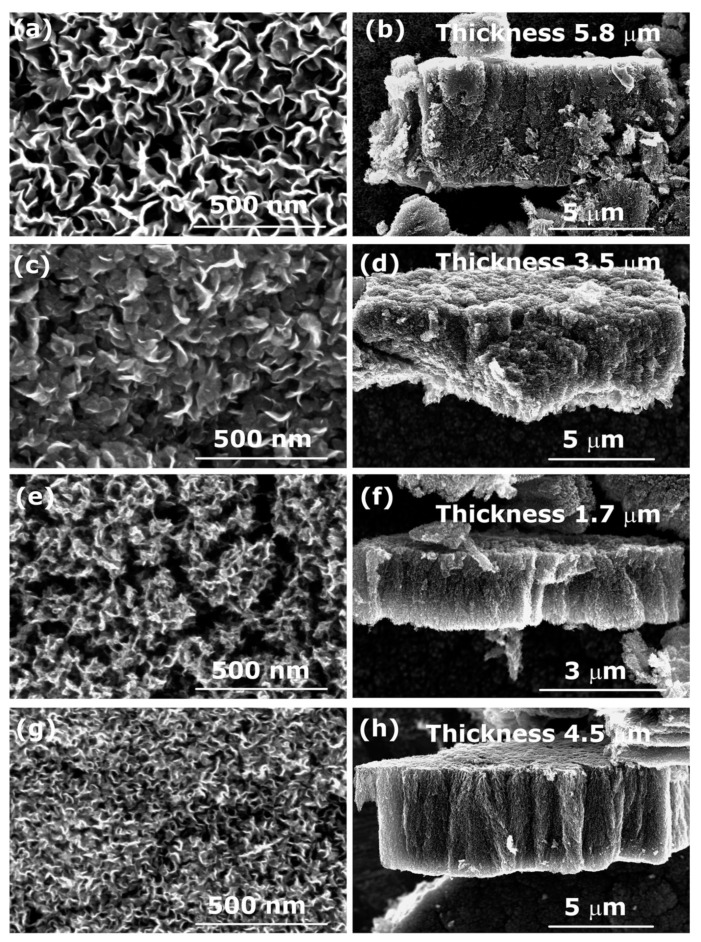
SEM images of carbon nanostructures (left) and their cross-section (right) synthesized from the aromatic PS polymer in plasma created in: (**a**,**b**) O_2_, (**c**,**d**) H_2_, (**e**,**f**) N_2_, and (**g**,**h**) CO_2_ gas. Deposition time was 60 s.

In order to check if this is true also for aromatic polymers, we show in Figure 8a–h SEM images of CNWs synthesized from PS polymer using different gases. Opposite to aliphatic polymer used in Figure 7, we can now observe the best formation of CNWs in the case of O_2_ plasma. It is known that O_2_ plasma treatment of aromatic polymers causes destruction and opening of the aromatic rings and thus a loss of aromatic structure [34,35], which can explain why we can only get CNWs in the case of O_2_ plasma treatment.

The method for synthesizing carbon nanowalls presented in this paper enables deposition rates as large as a few 100 nm/s. This is much larger compared to the classical technique using gaseous precursors. The deposition rates reported by various authors were presented in the review paper [7]. The achievable deposition rates were between about 0.1 and 100 nm/s, but most authors reported values between 1 and 10 nm/s. The highest deposition rate (300 nm/s), which deviates greatly from other reported data, was obtained by Zhang et al. [36], who used a mixture of argon, hydrogen, and methane at the pressure of 800 Pa, a discharge power of 18 kW, using a combination of inductively and capacitively coupled discharges. All other authors reported values below about 10 nm/s.

## 4. Conclusions

CNW growth in low-pressure plasma sustained by an inductively coupled RF discharge at the power of about 500 W was investigated using various polymers as solid precursors as well as various gases. The substrates were titanium mounted inside the RF coil and left at the floating potential. Important findings were derived from these investigations. We found that aromatic precursors were unsuitable for CNW growth, indicating that aromatic rings are not the most useful building blocks for CNWs. Aliphatic precursors which thermally degrade to simple low-weight C_x_H_y_ species were found more relevant for CNW growth. These two findings are in agreement with the generally accepted theory that C_2_ dimers are the most important building blocks involved in the formation of CNWs. In the presence of appropriate building blocks, CNWs will grow in plasma regardless of the gas type used for creating the discharge. The results indicate that the procedure elaborated in this paper may be applicable to mass production, as the optimal growth rate of a film consisting of good quality CNWs is over 100 nm/s.

## Figures and Tables

**Table 1:** Chemical structure of the polymers used as a carbon precursor.

Polymer	Structure
PS	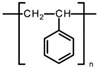
LDPE/HDPE	–CH_2_–CH_2_–
PP	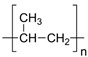
PET	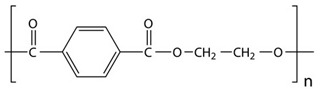
PA6	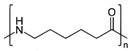
ABS	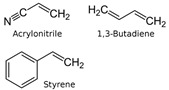

**Table 3:** XPS surface composition of the deposits synthesized in nitrogen plasma using various polymer sources.

Polymer	Gas	C (atom. %)	N (atom. %)	O (atom. %)	N/C
PS	N_2_	93.8	3.7	2.6	0.04
PET	N_2_	94.4	2.6	3.0	0.03
ABS	N_2_	98.0	0.7	1.3	0.03
PA6	N_2_	94.4	2.8	2.8	0.003
LDPE	N_2_	98.6	0.3	1.1	0.02
HDPE	N_2_	95.6	1.7	2.8	0.01
PP	N_2_	96.7	1.0	2.3	0.01

**Table 4:** XPS surface composition of the deposits synthesized from PP polymer using various gaseous discharges.

Polymer	Gas	C (atom. %)	N (atom. %)	O (atom. %)
PP	N_2_	96.7	1.0	2.3
PP	O_2_	97.5		2.5
PP	H_2_	98.3		1.7
PP	CO_2_	98.5		1.5

## Data Availability

The data presented in this study are available on request from the corresponding author.

## References

[1] Li J.H., Zhu M.J., An Z.L., Wang Z.Q., Toda M., Ono T. (2018). Constructing in-chip micro-supercapacitors of 3d graphene nanowall/ruthenium oxides electrode through silicon-based microfabrication technique. J. Power Sources.

[2] Liu L.L., Guan T., Fang L., Wu F., Lu Y., Luo H.J., Song X.F., Zhou M., Hu B.S., Wei D.P. (2018). Self-supported 3d nico-ldh/gr composite nanosheets array electrode for high-performance supercapacitor. J. Alloys Compd..

[3] Shin S.C., Yoshimura A., Matsuo T., Mori M., Tanimura M., Ishihara A., Ota K., Tachibana M. (2011). Carbon nanowalls as platinum support for fuel cells. J. Appl. Phys..

[4] Krivchenko V.A., Itkis D.M., Evlashin S.A., Semenenko D.A., Goodilin E.A., Rakhimov A.T., Stepanov A.S., Suetin N.V., Pilevsky A.A., Voronin P.V. (2012). Carbon nanowalls decorated with silicon for lithium-ion batteries. Carbon.

[5] Takeuchi W., Kondo H., Obayashi T., Hiramatsu M., Hori M. (2011). Electron field emission enhancement of carbon nanowalls by plasma surface nitridation. Appl. Phys. Lett..

[6] Wei W., Hu Y.H. (2017). Highly conductive na-embedded carbon nanowalls for hole-transport-material-free perovskite solar cells without metal electrodes. J. Mater. Chem. A.

[7] Vesel A., Zaplotnik R., Primc G., Mozetič M. (2019). Synthesis of vertically oriented graphene sheets or carbon nanowalls—Review and challenges. Materials.

[8] Vesel A., Zaplotnik R., Primc G., Mozetič M. (2020). A review of strategies for the synthesis of n-doped graphene-like materials. Nanomaterials.

[9] Vesel A., Zaplotnik R., Primc G., Pirker L., Mozetič M. (2021). One-step plasma synthesis of nitrogen-doped carbon nanomesh. Nanomaterials.

[10] Arnold C. (1979). Stability of high-temperature polymers. J. Polym. Sci. Macromol. Rev..

[11] Vohlídal J. (2021). Polymer degradation: A short review. Chem. Teach. Int..

[12] Dorai R., Kushner M.J. (2003). A model for plasma modification of polypropylene using atmospheric pressure discharges. J. Phys. D Appl. Phys..

[13] Zhang M., Buekens A., Jiang X., Li X. (2015). Dioxins and polyvinylchloride in combustion and fires. Waste Manag. Res..

[14] Peterson J.D., Vyazovkin S., Wight C.A. (2001). Kinetics of the thermal and thermo-oxidative degradation of polystyrene, polyethylene and poly(propylene). Macromol. Chem. Phys..

[15] Gewert B., Plassmann M.M., MacLeod M. (2015). Pathways for degradation of plastic polymers floating in the marine environment. Environ. Sci. Process. Impacts..

[16] Witkowski A., Stec A.A., Hull T.R., Hurley M.J., Gottuk D., Hall J.R., Harada K., Kuligowski E., Puchovsky M., Torero J., Watts J.M., Wieczorek C. (2016). Thermal decomposition of polymeric materials. Sfpe Handbook of Fire Protection Engineering.

[17] Samperi F., Puglisi C., Alicata R., Montaudo G. (2004). Thermal degradation of poly(ethylene terephthalate) at the processing temperature. Polym. Degrad. Stab..

[18] Holland B.J., Hay J.N. (2002). The thermal degradation of pet and analogous polyesters measured by thermal analysis–fourier transform infrared spectroscopy. Polymer.

[19] Davis R.D., Gilman J.W., VanderHart D.L. (2003). Processing degradation of polyamide 6/montmorillonite clay nanocomposites and clay organic modifier. Polym. Degrad. Stab..

[20] Teii K., Shimada S., Nakashima M., Chuang A.T.H. (2009). Synthesis and electrical characterization of n-type carbon nanowalls. J. Appl. Phys..

[21] Yu K.H., Wang P.X., Lu G.H., Chen K.H., Bo Z., Chen J.H. (2011). Patterning vertically oriented graphene sheets for nanodevice applications. J. Phys. Chem. Lett..

[22] Meško M., Vretenár V., Kotrusz P., Hulman M., Šoltýs J., Skákalová V. (2012). Carbon nanowalls synthesis by means of atmospheric dcpecvd method. Phys. Status Solidi B.

[23] Teii K., Hori M., Goto T. (2001). Negative bias dependence of sulfur and fluorine incorporation in diamond films etched by an sf_6_ plasma. J. Electrochem. Soc..

[24] Lehmann K., Yurchenko O., Urban G. (2016). Effect of the aromatic precursor flow rate on the morphology and properties of carbon nanostructures in plasma enhanced chemical vapor deposition. RSC Adv..

[25] Hsu C.-C., Bagley J.D., Teague M.L., Tseng W.-S., Yang K.L., Zhang Y., Li Y., Li Y., Tour J.M., Yeh N.C. (2018). High-yield single-step catalytic growth of graphene nanostripes by plasma enhanced chemical vapor deposition. Carbon.

[26] Ostrikov K., Neyts E.C., Meyyappan M. (2013). Plasma nanoscience: From nano-solids in plasmas to nano-plasmas in solids. Adv. Phys..

[27] Suzuki M., Wilkie C.A. (1995). The thermal degradation of acrylonitrile-butadiene-styrene terpolymei as studied by tga/ftir. Polym. Degrad. Stab..

[28] Lee K.-H., Shin D.-H., Seo Y.-H. (2006). Thermal degradation of nitrogen-containing polymers, acrylonitrile-butadiene-styrene and styrene-acrylonitrile. Korean J. Chem. Eng..

[29] Hiramatsu M., Kondo H., Hori M., Gong J.R. (2013). Graphene nanowalls. New Progress on Graphene Research.

[30] Cui L., Chen J., Yang B., Sun D., Jiao T. (2015). Rf-pecvd synthesis of carbon nanowalls and their field emission properties. Appl. Surf. Sci..

[31] Jiang L., Yang T., Liu F., Dong J., Yao Z., Shen C., Deng S., Xu N., Liu Y., Gao H.-J. (2013). Controlled synthesis of large-scale, uniform, vertically standing graphene for high-performance field emitters. Adv. Mater..

[32] Wang J., Zhu M., Outlaw R.A., Zhao X., Manos D.M., Holloway B.C. (2004). Synthesis of carbon nanosheets by inductively coupled radio-frequency plasma enhanced chemical vapor deposition. Carbon.

[33] Hiramatsu M., Hori M. (2010). Carbon Nanowalls: Synthesis and Emerging Applications.

[34] Gonzalez E., Barankin M.D., Guschl P.C., Hicks R.F. (2009). Ring opening of aromatic polymers by remote atmospheric-pressure plasma. IEEE Trans. Plasma Sci..

[35] Liang Y., Li J., Xue Y., Tan T., Jiang Z., He Y., Shangguan W., Yang J., Pan Y. (2021). Benzene decomposition by non-thermal plasma: A detailed mechanism study by synchrotron radiation photoionization mass spectrometry and theoretical calculations. J. Hazard. Mater..

[36] Zhang H., Wu S., Lu Z., Chen X., Chen Q., Gao P., Yu T., Peng Z., Ye J. (2019). Efficient and controllable growth of vertically oriented graphene nanosheets by mesoplasma chemical vapor deposition. Carbon.

